# Stability and change in screen-based sedentary behaviours and associated factors among Norwegian children in the transition between childhood and adolescence

**DOI:** 10.1186/1471-2458-12-104

**Published:** 2012-02-06

**Authors:** Mekdes K Gebremariam, Torunn H Totland, Lene F Andersen, Ingunn H Bergh, Mona Bjelland, May Grydeland, Yngvar Ommundsen, Nanna Lien

**Affiliations:** 1 Department of Nutrition, Faculty of Medicine, University of Oslo, PO Box 1046 Blindern, N-0316 Oslo, Norway; 2 Department of Coaching and Psychology, Norwegian School of Sport Sciences, Oslo, Norway; 3 Department of Sports Medicine, Norwegian School of Sport Sciences, Oslo, Norway

## Abstract

**Background:**

In order to inform interventions to prevent sedentariness, more longitudinal studies are needed focusing on stability and change over time in multiple sedentary behaviours. This paper investigates patterns of stability and change in TV/DVD use, computer/electronic game use and total screen time (TST) and factors associated with these patterns among Norwegian children in the transition between childhood and adolescence.

**Methods:**

The baseline of this longitudinal study took place in September 2007 and included 975 students from 25 control schools of an intervention study, the HEalth In Adolescents (HEIA) study. The first follow-up took place in May 2008 and the second follow-up in May 2009, with 885 students participating at all time points (average age at baseline = 11.2, standard deviation ± 0.3). Time used for/spent on TV/DVD and computer/electronic games was self-reported, and a TST variable (hours/week) was computed. Tracking analyses based on absolute and rank measures, as well as regression analyses to assess factors associated with change in TST and with tracking high TST were conducted.

**Results:**

Time spent on all sedentary behaviours investigated increased in both genders. Findings based on absolute and rank measures revealed a fair to moderate level of tracking over the 2 year period. High parental education was inversely related to an increase in TST among females. In males, self-efficacy related to barriers to physical activity and living with married or cohabitating parents were inversely related to an increase in TST. Factors associated with tracking high vs. low TST in the multinomial regression analyses were low self-efficacy and being of an ethnic minority background among females, and low self-efficacy, being overweight/obese and not living with married or cohabitating parents among males.

**Conclusions:**

Use of TV/DVD and computer/electronic games increased with age and tracked over time in this group of 11-13 year old Norwegian children. Interventions targeting these sedentary behaviours should thus be introduced early. The identified modifiable and non-modifiable factors associated with change in TST and tracking of high TST should be taken into consideration when planning such interventions.

## Background

Increases in sedentary behaviours (SB), and in particular entirely sedentary pastimes, such as television (TV) viewing and use of computer/electronic games have been reported over the past decades [1,2]. High levels of SB have adverse impacts on child and adult health. In young people, SB has been found to be associated with increased body weight and obesity [2-8], adverse metabolic profiles [9], and poor fitness in later life [3]. Although these associations are complex and have variable magnitudes [10], they do suggest that interventions targeting SB in children and adolescents are of public health importance. In addition, studies have shown that reducing SB without specifically targeting physical activity (PA) can increase levels of PA [11,12], which is important since PA has several short and long term benefits for the well-being of children and adolescents [13-15]. Nevertheless, SB seems more inherently reinforcing and has been found to be more difficult to change than PA in this age group [11].

Interventions targeting SB are likely to be more successful if implemented in developmental periods during which stability of the behaviours is relatively low [16]. Available data suggests that TV viewing and use of computer/games are relatively stable behaviours which track moderately in childhood and adolescence [10]. Tracking refers to the tendency of individuals to maintain their rank or position within a group over time [17] and to the ability to predict future values based on previous values [18]. Studying tracking patterns of screen time is important to see the stability and change of SB over time. It can help determine the proper timing of interventions targeting these behaviours. It is also vital to assess factors associated with screen time tracking patterns, as well as correlates of change in screen time in order to inform such interventions [19]. Whereas modifiable correlates can be directly addressed, non- modifiable correlates could indicate particular risk groups which should be targeted in interventions. However, few longitudinal studies have looked at screen-based SB and their changes over time. A recent review concluded that prospective studies of SB in young people are scarce; and documented insufficient evidence for determinants of SB from the included prospective studies [20]. In addition, most available studies have focused on TV viewing, ignoring other screen time behaviours which can account for a significant portion of time spent sedentary [21,22]. By focusing on a single screen time behaviour one is unlikely to capture the diversity of leisure behaviour in young people [19], and thus other screen time behaviours such as electronic game/computer use and total screen time (TST) should also be looked into. A need for more studies looking at these behaviours has been emphasized [10,23].

This paper aims to assess stability (tracking) and changes of TV/DVD, computer/electronic game use and TST over a 2 year period, and to assess variables associated with change in TST and with tracking high (vs low) TST in a group of Norwegian children in the transition between childhood and adolescence.

## Methods

### Design and sample

This study makes use of data from a school-based intervention, the HEalth In Adolescents (HEIA) study [24]. The overall aim of the HEIA study was to develop and evaluate a multi-component intervention study aimed at healthy weight development through diet and physical activity. A total of 177 schools were invited, and 37 schools accepted the invitation. Schools were included in this study if they had a minimum of 40 enrolled pupils in the 6th grade. Schools were thus recruited from the largest towns/municipalities in seven counties from the Eastern part of Norway (Akershus, Buskerud, Hedmark, Oppland, Telemark, Vestfold, and Østfold). The schools were randomly assigned into 12 intervention and 25 control schools. All 6th graders in these schools and their parents/legal guardians were invited to participate in the baseline (BL) study which took place in September 2007.

The participants in the present study are students from the 25 control schools. Parental consent was obtained for 1014 children from these schools, with a rate of parental consent of 73%. A total of 975 students (96% of the 1014 returning parental informed consent) from these schools participated at baseline (BL). The first follow-up (T1) study took place in May 2008, and 934 (96%) of those participating at BL participated. The second follow-up (T2) was conducted in May 2009, and 885 (95%) of those participating at BL and T1 participated.

Ethical clearance was obtained from the Regional Committees for Medical Research and the Norwegian Social Science Data Service.

### Data collection

The children answered an internet-based questionnaire over a period of approximately 45 minutes at all time points. The children were taken to separate computer rooms in groups. Research assistants were present to answer questions, resolve technical problems and ensure that children replied independently from each other.

### Measures

Four questions with pre-coded answer categories were asked to assess usual TV/DVD use and use of computer/electronic games: How many hours do you usually watch TV and/or DVD on a normal weekday? The same question was asked for a normal weekend day. The answer categories were (recoding in brackets): half hour [0.5], one hour [1], two hours [2], three hours [3], four hours [4], five hours or more [5]. The two questions on computer/electronic game use were formulated in the same way as for TV/DVD, but the answer categories were: no playing [0], half hour or less [0.5], one hour [1], two hours [2], three hours [3], four hours or more [4]. Separate weekly scores for TV/DVD and computer/electronic games were calculated by summing hours reported for an average weekday (multiplied by five) and average weekend day (multiplied by 2), and then summed to create a total screen time (TST) variable.

Moderate test-retest correlation coefficients (0.66-0.73) for the weekly outcome measures were obtained from a separate test-retest study conducted at 10-14 days apart among 111 6th graders prior to the main data collection.

#### Independent variables

Perceived parental regulation of TV/DVD use and perceived parental regulation of computer/electronic games were assessed by a 4-item scale (e.g. My mother and father try to make sure that I do not watch too much TV; my mother and father try to make sure that I do not use the computer and play games too much) [25]. The items in these measures were rated on a 5-point Likert-type scale coded 1 (lowest) to 5 (highest) and phrased "totally disagree" to "totally agree" with a neutral midpoint. Since the outcome measure for the regression analyses was TST, all items in both measures were used to form a parental TST regulation measure. Reliability analysis was done for this measure, and a high internal consistency was obtained: Cronbach's α = 0.80 (BL), 0.85 (T2).

Self-efficacy related to barriers for PA was assessed by a 5-item scale, adapted and modified from Motl et al. 2006 [26] and Lytle 2009 [27] (e.g. I can be physically active during my free time on most days even if I have the choice to watch TV or play video games instead). Items of the scale were rated on a 5-point Likert-type scale. The scale had high internal consistency with Cronbach's α = 0.75 (BL) and 0.77 (T2).

BMI was calculated as weight/height^2^. Weight and height were objectively measured. The age and gender specific BMI cut-off values proposed by the International Obesity Task Force [28] were used in order to categorize the adolescents into non-overweight and overweight/obese.

The pubertal development scale is based on the pubertal category scores defined by Carskadon and Acebo [29]. The categories were: prepubertal, early pubertal, mid-pubertal, late pubertal or post-pubertal (re-categorization into pre-pubertal/early pubertal/mid, late and post-pubertal was done for the analyses).

Participants were divided into either ethnic Norwegian or ethnic minority. Ethnic minorities are defined as those having both parents born in a country other than Norway [30], and constituted 6.5% of the total sample.

Living status of children was divided into two categories: those living with married or cohabitating parents constituted the first group; those living with their father or mother alone, equally with their mother or father, grandparents or another adult were grouped together in the second group.

Parental education was gathered as part of the parental informed consent for the adolescent. It was categorized into: low (12 years or less), medium (between 13 and 16 years) and high (more than 16 years). Educational status of the parent with the longest education or else the one available was used in the analyses.

### Statistical analysis

Since schools were the unit of measurement in this study, we checked for clustering effect through the Linear Mixed Model procedure. Only 1.4-3.2% of the unexplained variance in the variables investigated was at the school level. Hence, adjustment for clustering effect was not conducted.

Mean values (standard deviations) were calculated for the outcome measures at baseline, T1 and T2, as these are approximately normally distributed. Gender-specific tertiles were computed from the average weekly TV/DVD use, computer/electronic game use and TST. The TST was also categorized into two, representing those with a weekly TST of less than 14 hours and those with a weekly TST of more than 14 hours. This cut-off was chosen based on recommendations for a maximum total daily electronic media use time of 2 hours or less in some countries [31,32].

Analysis of variance (ANOVA) was conducted to assess differences between males and females in mean levels of weekly hours spent watching TV/DVD, weekly hours spent using computer/electronic games, and mean hours of weekly TST at the three time points, and one-way repeated measures ANOVA was used to compare time spent on these SB over the three time points. ANOVA was also used to evaluate associations between TST and different characteristics of participants at BL and T2.

Tracking was examined using Generalized Estimating Equations (GEE), one of the latest statistical methods used for this purpose. GEE analysis has several advantages: it allows for all longitudinal data to be used to calculate a single stability coefficient, taking into account that repeated measurements within an individual are correlated; it also allows for adjustment for both time dependent and time independent covariates [18]. In this method of analysis, the value of the initial measurement of the outcome variable at time 1 is regressed on the longitudinal development of the outcome variable from time 2 to time m (where m = number of measurements), adjusting for covariates. This allows for the assessment of the relationship between the first measurement and subsequent measurements, yielding one single regression coefficient, the standardized value of which can be interpreted as a longitudinal correlation coefficient [18]. In this study, adjustment for ethnicity, parental education, family status and weight status was done. Correlation coefficients of < 0.30 were classified as low, 0.30 to 0.60 as moderate, and > 0.60 as moderately high [33]. Then, to measure level of agreement between tertile membership at BL and T2, Cohen's weighted kappa was computed. According to Landis and Koch [34], values of 0.01 to 0.2 indicate slight agreement, 0.21 to 0.40 fair agreement, 0.41 to 0.60 moderate agreement, 0.61 to 0.80 substantial agreement and 0.81 to 1.00 almost perfect agreement. Direct weighted Kappa calculation is not possible on SPSS, thus syntax from the IBM website [35] was used. Data for imputation into the syntax was obtained from cross-tabulation.

Linear regression analysis was conducted to assess factors associated with change in TST between BL and T2, after correcting for baseline TST. The BL variables included in the regression analysis were perceived parental regulation of screen time, self-efficacy related to barriers for PA, BMI category, pubertal stage, ethnicity, living status and parental education.

Multinomial logistic regression analysis was then used to assess whether these BL characteristics of participants could be predictors of tracking high (vs low) TST. Subgroup analysis was done for this purpose including those in the lower and upper tertile at BL (low and high users). Then, three tracking patterns were identified: tracking high TST (those in the upper tertile for TST at BL who remained there at T2, or high-risk group), tracking low TST (those in the lower tertile for TST at BL who remained there at T2, or low-risk group) and no tracking of TST (increase or decrease).

Both univariate and multivariate analyses were conducted. Selection of variables for inclusion in the multivariate models was based on the results of univariate analysis (*p *≤ 0.25). Results of the final adjusted models (unadjusted regression coefficients or odds ratios and 95% CI) are presented in text.

TST was used as an outcome variable instead of TV/DVD and computer/game use separately in the regression analyses. A review study has indicated that correlates of separate screen time behaviours are similar to those of overall screen viewing in younger children, with few exceptions [36], and separate analysis (data not shown) showed that it was the case in this study as well. In addition, interventions are most likely to target overall screen time behaviours and not separate ones.

Attrition analysis was performed using independent samples *t*-test and chi-squared test of proportions to determine differences in baseline characteristics between participants and drop-outs (n = 90).

All statistical analyses were performed by IBM^® ^SPSS^® ^Statistics, version 18.0 (IBM Corp., Somers, New York, USA).

## Results

### Descriptives and change

A total of 885 students (47% female and 53% male) who participated at all three time points were included in the analysis. The mean age at baseline was 11.2 (0.3).

The mean weekly hours spent on TV/DVD use, computer/game use, and TST at BL, T1 and T2 for boys, girls and the total sample are presented in Table 1. The increases in time spent weekly on these behaviours between BL and T2 were statistically significant (*p *< 0.01). There was also a significant increase in the screen time behaviours between the different time points, except for computer/game use among males between BL and T1 (*p *= 0.174) and TV viewing for males between T1 and T2 (*p *= 0.312).

**Table 1:** Sedentary behaviors, mean values and proportions at baseline, 1 and 2 year follow-up among Norwegian children

		BL		T1		T2	
		
		Mean	SD	Mean	SD	Mean	SD
TV/DVD use	Female	11.04	6.52	12.36	6.62	13.45	6.86

	Male	11.90	6.77	12.94	7.25	13.28	7.37

	Total	11.50	6.66	12.66	6.96	13.36	7.13

Computer/electronic game use	Female	7.31*	5.81	8.00*	5.55	9.88^#^	6.82

	Male	9.80*	6.63	10.19*	6.84	11.01^#^	7.37

	Total	8.61	6.37	9.15	6.36	10.47	7.14

Total screen time	Female	18.44*	10.91	20.34*	9.88	23.31	11.34

	Male	21.68*	11.83	23.14*	12.05	24.30	12.73

	Total	20.15	11.51	21.81	11.15	23.83	12.10

		%		%		%	

Total screen time > 2 hrs/day	Female	50.7		67.1		72.7	

	Male	65.9		73.9		73.4	

	Total	58.7		70.6		73.1	

*, ^#^statistically significant difference between boys and girls, *: *p *< 0.001, ^#^: *p *< 0.05

N = 419 for girls and 466 for boys and varies slightly between variables

Statistically significant changes between BL and T2 for all variables

There was no significant difference in TV/DVD watching between boys and girls at any time point, but there was a significant difference in computer/game use at all time points, with boys spending more time on computer/game use than girls There was a significant difference between boys and girls in TST at BL and T1 (*p *< 0.001), but not at T2 (*p *= 0.226).

The mean change in TST between BL and T2 was 4.98 hrs/week for girls and 2.73 hrs/week for boys (*p *= 0.007).

The proportion of children with a daily TST of over 2 hours increased from 50.7% at BL to 67.1% at T1 to 72.7% at T2 among girls, and from 65.9% at BL to 73.9% at T1 to 73.4% at T2 among boys.

The attrition analysis showed that there was no significant difference in the outcome measures and the independent variables at BL between participants and drop-outs.

### Tracking

Stability coefficients based on GEE analysis showed statistically significant (*p *< 0.001) moderate correlations. For females, coefficients of 0.48, 0.38 and 0.48 were obtained for TV/DVD use, computer/game use and TST. The respective coefficients for males were 0.47, 0.49 and 0.51 (Table 2). Tracking coefficients for TST and TV/DVD use were similar between genders. Tracking of computer/electronic games was slightly higher in boys than girls.

**Table 2:** Stability coefficients for sedentary behaviors among Norwegian 11-13 year-old children

	Girls	Boys
TV/DVD	0.48 (0.41-0.55)	0.47 (0.38-0.55)

Electronic game/computer	0.38 (0.30-0.47)	0.49 (0.41-0.57)

Total screen time	0.48 (0.38-0.54)	0.51 (0.44-0.61)

Stability coefficients computed from GEE analysis

All coefficients are significant at the 0.001 level

Coefficients were adjusted for ethnicity, parental education, family status and weight status

The weighted kappa values also indicate the presence of fair to moderate tracking within tertiles. Results are presented in Table 3.

**Table 3:** Tracking patterns of sedentary behaviors between baseline and 2 year follow-up among Norwegian children

	Proportion of tracking*				
	
	Tertile 1**	Tertile2	Tertile3	Overall proportion of tracking***	Weighted ᵏ
TV/DVD use					

Girls	60	35	51	49	0.40

Boys	64	30	41	45	0.36

Electonic game/computer use					

Girls	47	40	52	46	0.28

Boys	51	40	61	50	0.39

Total screen time					

Girls	55	43	59	52	0.41

Boys	55	42	55	50	0.41

N = 419 girls and 466 boys, and varies slightly between variables

*Proportion of children who were in lower, middle and upper tertile at T2 who were there at baseline

**Lower tertile

***Or percentage of agreement

### Factors associated with an increase in TST between BL and T2

Table 4 shows factors associated with TST at BL and T2 in both genders. Analysis was done to assess whether these factors would be associated with changes in screen time between these two time points. In females, the only variable found to be associated with an increase in TST between BL and T2 in the multivariate analysis was high parental education (inverse) (B = -2.67 (-5.17,-0.18)), indicating an increase in TST of around 2.7 hours for those with low parental education. Among males, living with married or cohabitating parents (B = -3.09 (-5.69.-0.50)) and self-efficacy related to barriers to PA (B = -2.16 (-3.60,-0.73)) were inversely related to an increase in TST, indicating an increase in TST of around 3 hours per week for those not living with married or cohabitating parents and a decrease of around 2.2 hours per week per unit increase in self-efficacy score.

**Table 4:** Association between socio-demographic, psycho-social and biological characteristics of Norwegian children and total screen time

Characteristics of participants	Total screen time at baseline						Total screen time at T2					
	**Girls**			**Boys**			**Girls**			**Boys**		

	M*	SD*	**p**	M*	SD*	**p**	M*	SD*	**p**	M*	SD*	**p**

Self-efficacy related to barriers to physical activity												

Low	19.56	11.10	0.02	24.46	12.07	< 0.001	25.01	11.68	0.001	27.39	13.17	< 0.001

High	16.96	10.39		17.19	9.50		21.13	10.58		20.82	11.19	

Parental regulation of screen time												

Low	19.89	11.71	0.13	22.31	12.01	0.32	24.49	11.99	0.24	26.33	13.67	0.02

High	17.96	10.57		21.10	11.46		22.94	11.11		23.38	12.25	

BMI status												

Non-overweight	17.88	10.30	0.04	20.81	11.18	< 0.001	22.84	11.09	0.08	23.88	12.41	0.07

Overweight/obese	20.93	12.79		27.01	14.25		25.82	12.20		27.40	14.13	

Pubertal stage												

Pre-pubertal	16.47	10.30	0.40	21.61	10.30	0.74	20.88	10.29	0.29	22.68	12.22	0.32

Early pupertal	19.30	11.49		21.07	11.57		24.15	11.40		24.74	12.56	

Mid/late/post-pubertal	18.44	10.65		20.28	12.46		23.56	11.50		24.17	12.46	

Ethnicity												

Norwegian	17.79	10.10	< 0.001	21.65	11.78	0.87	23.10	11.14	0.25	24.37	12.76	0.78

Ethnic Minority	26.43	14.77		22.04	12.21		25.60	13.62		23.65	12.71	

Parental education												

Low	18.88	12.16	0.51	22.85	13.44	0.23	25.18	11.71	0.03	25.74	13.70	0.20

Medium	18.98	10.02		20.59	10.60		23.42	11.71		24.01	12.03	

High	17.59	10.72		21.05	10.84		21.52	10.43		23.06	12.54	

Living status												

Married or cohabitating parents	18.72	10.74	0.26	20.83	11.56	0.003	23.17	11.32	0.62	23.26	12.13	0.001

Other	17.13	11.64		24.97	12.45		23.89	11.47		28.15	14.30	

*Values presented as Mean (M) and Standard Deviations (SD), n = 419 girls and 466 boys, and varies slightly between variables

### Predictors of tracking of high TST

Results of the multinomial regression analysis show that, among girls, children with low self-efficacy related to barriers to PA were more likely to track high TST (OR = 2.30, C.I. = 1.13-4.69) compared to children with high self-efficacy; whereas girls who are ethnic Norwegian were less likely to track high TST (OR = 0.23, CI = 0.06-0.91) than the group of ethnic minority (Table 5). Among males, overweight/obese children were more likely to track high TST (OR = 4.58, CI = 1.49-14.03) compared to those not overweight. Similarly, boys who did not live with married or cohabitating parents were more likely to track high TST (OR = 3.10, CI = 1.32-7.30) compared to the group covering others. Boys with low self-efficacy related to barriers to PA were also more likely to track high TST (OR = 6.83, CI = 3.22-14.45) than the group with high self-efficacy.

**Table 5:** Factors associated with tracking high Total Screen Time (TV/DVD and electronic game/computer) among Norwegian children

	Tracking high total screen time		
**Baseline characteristics**	**OR**	**C.I**.	**p**

**Females**			

Self-efficacy related to barriers to PA			

Low	2.30	1.13-4.69	**0.022**

High	1		

Pubertal stage			

Pre-pubertal	0.36	0.10-1.32	0.123

Early pubertal	1.40	0.53-3.68	0.491

Mid/late/post-pubertal	1		

Parental education			

Low	1.43	0.58-3.52	0.430

Medium	1.49	0.63-3.50	0.364

High	1		

Ethnicity			

Ethnic Norwegian	0.23	0.06-0.91	**0.036**

Ethnic Minority	1		

**Males**			

Self-efficacy related to barriers to PA			

Low	6.83	3.22-14.45	**< 0.001**

High	1		

BMI status			

Overweight/obese	4.58	1.49-14.03	**0.008**

Not overweight	1		

Parental education			

Low	1.66	0.70-3.96	0.251

Medium	1.03	0.43-2.45	0.943

High	1		

Living status			

Other	3.10	1.32-7.30	**0.010**

Married or cohabitating Parents			

Variables entered in the final model of the multinomial logistic regression for both genders are shown

Tracking low TST was treated as the outcome reference category

## Discussion

This study investigated stability and changes in screen-based sedentary behaviours in a group of Norwegian children in a transition phase between childhood and adolescence, and factors associated with stability and change in total screen time.

Findings based on both absolute and rank measures indicate that the SB investigated had fair to moderate tracking between the beginning of 6th and the end of 7th grade. The tracking patterns identified in this study are similar to those reported for children in similar age groups and over similar periods [10], although comparability between tracking studies is complicated among other things by the duration of follow-up and by the method used to assess tracking including whether adjustment for potential covariates has been done or not. Tracking patterns were largely similar between boys and girls, except for a small difference in the tracking coefficients of electronic games/computer use. A review concluded that there seemed to be little evidence for any gender differences in tracking of SB [10], and our study supports this. The findings that boys and girls watch the same amount of TV and that boys use more electronic games/computers than girls have similarly been documented in the literature previously [19,37,38].

There was an increase in time spent on both SB during the studied period, but TV viewing seemed to start levelling off in boys. Similarly, over 70% of both boys and girls exceed the recommendations in some countries for a maximum daily total daily electronic media use time of 2 hours or less [31,32]. These findings are in line with the reported increases in SB and in particular electronic media use that occurs in childhood and early adolescence [1,2]. Accessibility to SB seems to be easier than that to PA in most people [11], and this accessibility might increase as the children grow older because they spend more time alone at home and may also have TVs and computers in their own rooms. These factors might further increase screen time.

Higher screen time at BL and higher odds of tracking high TST among girls with an ethnic minority background compared to ethnic Norwegian girls might be due to factors such as low integration as well as cultural differences which might make other recreational activities less common among ethnic minority girls. A study among adolescents has previously shown that girls with an ethnic minority background are likely to be more inactive than ethnic Norwegian girls, whereas no difference was found in boys [39]. Parental education was also found to be inversely associated with TST among girls. The inverse relationship between parental education and use of electronic media has previously been documented [19,40,41].

Not living with married/cohabitating parents was positively associated with an increase in TST, and with tracking high TST among males. A review of correlates of TV viewing among youth concluded that young people in single-parent/guardian families consistently watch more TV than those from two parent/guardian families [19]. Overweight/obese boys in the study were also more likely to tracking high TST compared to those not overweight. This might indicate a bi-directional relationship: high TST might influence children's BMI and high BMI might lead to children reverting to more SBs. As mentioned earlier, several studies have shown an association between screen time behaviours and in particular TV use and being overweight [2-8]. One possible explanation for the presence of this association in boys only in this study might be the fact that boys engage in leisure time PA more often than girls and they also tend to engage in more strenuous exercises and focus more on achievement [42]. Thus, overweight/obese boys might want to withdraw from such PA and instead revert to sedentary leisure time activities such as screen time activities because of potential physical limitations as well as psychological fears of lack of competence to engage in such PAs. Other studies have similarly found an association in male adolescents and none among their female counterparts [43], in line with the findings from this study.

A variable consistently inversely associated with TST in both genders was self-efficacy related to barriers to PA, one barrier being screen time itself. Self-efficacy related to barriers to PA was investigated for its association with SB in this study because it is the strongest and most consistent correlate of PA in this age group [44,45], also found previously in the HEIA study [46]. The displacement hypothesis suggests that SB can displace time that could otherwise have been used for PA [47]. Findings from several studies do not support this hypothesis, as indicated by a large meta-analysis [5]. Negative associations between SB and PA have nevertheless been documented [12,16,47]. Self-efficacy related to barriers to PA, by positively influencing levels of PA, can consequently potentially lead to decreased time spent on SB, which appears to be the case in this study. It has been suggested that targeting self-efficacy should have a prominent position in efforts to promote PA [45]. The results of this paper suggest that such efforts might also be successful in lowering time spent on electronic media use.

It has been suggested that SB might be more strongly associated with socio-demographic factors than modifiable factors such as psychosocial or behavioural variables [19,40]. It is nevertheless possible that variables from the psychological and behavioural domains play a greater role in influencing TST in this age group of children, and even mask the importance of socio-demographic factors. There is thus a need to look into such correlates of TST in children in this age group.

It is not likely that there was any difference in tracking and change patterns between participants and drop-outs as the attrition analyses did not show any difference in baseline characteristics between these two groups.

### Strengths and weaknesses

This was a longitudinal study with a relatively large sample size. In addition, the rate of retention in this study was very high. The study also provides much needed information about screen time patterns in a Norwegian context. It is also one of a few studies looking at factors associated with tracking patterns of TST. Different screen time behaviours were included. Gender-related differences as well as similarities were also reflected.

The use of self-reported measures is associated with problems of validity and reliability, in particular in younger children. Nevertheless, there was a good test-retest reliability of the outcome measures. In addition, the trends in screen time obtained in this study were found to be in line with those found in other studies, including the gender differences noted [19,37,38]. The study looked at stability and change in screen time behaviours, as well as associated factors and comparisons between genders, and we believe that the measures used are reasonably reliable and valid for such purposes. In general, the more unreliable the measures, the higher the chance of Type II errors; that is not finding differences and associations that in fact exist. Therefore, differences and associations in the study were probably underestimated rather than overestimated.

Finally, the study is restricted to a single geographical area, therefore generalizability is limited.

## Conclusion

In a period of rapid behavioural, psychological and biological development, the sedentary behaviours investigated in this study were found to have fair to moderate tracking. With few exceptions, tracking patterns appear to be similar between boys and girls. Since interventions are likely to be more successful if implemented in developmental periods during which stability of behaviour is relatively low [16], younger age groups need to be targeted for interventions aimed at lowering electronic media use in this setting. Such interventions, if successful are also likely to have long-lasting effects.

The identified modifiable correlates of TST and tracking of high TST, self-efficacy related to barriers to physical activity in both genders and weight status in males, need to be directly targeted. The non-modifiable correlates comprising ethnicity and parental education in females and living status in males, give a hint as to which groups should be particularly targeted for intervention, whenever feasible.

## Competing interests

The authors declare that they have no competing interests.

## Authors' contributions

M.K.G. conducted the statistical analyses and wrote the first draft of the manuscript and made the greatest contribution to the paper. N.L., L.F., I.H.B, M.B., M.G. and YO participated in designing the study, project planning and data collection. All authors have critically revised the manuscript, and read and approved the final version of the manuscript.

## Pre-publication history

The pre-publication history for this paper can be accessed here:

http://www.biomedcentral.com/1471-2458/12/104/prepub
